# Carbon monoxide: a critical physiological regulator sensitive to light

**DOI:** 10.1038/s41398-020-0766-1

**Published:** 2020-03-09

**Authors:** Dan A. Oren, Dorothy K. Sit, Sohrab H. Goudarzi, Katherine L. Wisner

**Affiliations:** 1 Department of Psychiatry, Yale University, New Haven, CT USA; 2 Department of Psychiatry, Northwestern University, Chicago, IL USA; 3 KCAS Bioanalytical and Biomarker Services, Shawnee, KS USA

**Keywords:** Biomarkers, Physiology

## Abstract

The mechanism by which humans absorb therapeutic light in winter seasonal and nonseasonal depression is unknown. Bright-light-induced release and generation of blood-borne gasotransmitters such as carbon monoxide (CO) may be one mechanism. Here, 24 healthy female volunteers had peripheral blood samples drawn. Samples were collected in a dimly lit room and protected from light exposure. Samples were analyzed for CO concentrations by gas chromatography after 2 h of continuous exposure to darkness vs. bright white light. In a similar confirmatory study, 11 additional volunteers had samples analyzed for CO concentrations after 2 h of continuous exposure to gentle rocking in darkness vs. in bright white light. In the first study, light-unexposed peripheral blood had a mean CO concentration of 1.8 ± 0.4 SD ppm/g. Identically treated samples with 2 h of rocking and exposure to bright white light at illuminance 10,000 lux had a mean CO of 3.6 ± 1.2 ppm/g (*p* < 0.0001). Post hoc analysis of that study showed that time of day was significantly inversely associated with increase in CO concentration under bright light vs. dark (*p* < 0.04). In a smaller confirmatory study of 11 healthy female volunteers, after 2 h of rocking, light-unexposed peripheral blood had a mean CO of 1.4 ± 0.5 SD ppm/g. Identically treated blood samples with 2 h of exposure to bright white light at illuminance 10,000 lux had a mean CO of 2.8 ± 1.7 ppm/g (*p* < 0.02). In conclusion, bright-light exposure robustly increases human blood CO in vitro. This supports the putative role of CO as a physiological regulator of circadian rhythms and light’s antidepressant effects. This human evidence replicates earlier data from a preclinical in vivo model. This effect may be stronger in the morning than in the afternoon.

## Introduction

Bright-light therapy is one of the best-studied nonpharmacological treatments for depressive disorders^1,2^. The molecular mechanism by which humans absorb light that has energizing and antidepressant effects in winter seasonal (seasonal affective disorder (SAD))^3^ and nonseasonal depression^4^ and bipolar depression^5^ is unknown. Studies in humans and animals suggest that the antidepressant effect is mediated through light exposure to the eyes.

A recent study in nocturnally active rodents suggests that intrinsically photosensitive retinal ganglion cells, absorbing light via the retinal photopigment melanopsin, directly mediate light’s effects upon mood and learning via the perihabenular nucleus of the thalamus^6^. The applicability of these findings to diurnally active humans remains to be demonstrated and does not preclude the possibility of alternative or complementary pathways of antidepressant light absorption.

Though Darwin reported in 1880 that “hardly anyone supposes that there is any real analogy between the sleep of animals and that of plants^7^,” seasonal and circadian behaviors of plants have been observed for millennia. The response of many biological rhythms to manipulations of ambient light in animals strikingly resembles responses in plants. Such phenomena in plants and animals, in the lab and in the field, can be recreated by properly timed exposure to bright light and darkness. For a patient with winter depression successfully treated with bright light or by the natural arrival of springtime, the improvement in mood and energy experienced bears at least superficial parallels with the routine blooming of plant life in spring and summer.

In this context, we can ask whether molecular mechanisms of chronobiological light absorption and light-driven seasonal changes might be conserved across the plant and animal kingdoms. Despite the vast differences in plant and animal biology, increasing evidence of common aspects of plant and animal behavior and plant and animal sensory mechanisms is being discovered^8^.

An evolutionary-based model of “humoral phototransduction” draws upon the common biosynthetic pathways of the chromophores of chlorophyll in plants and heme molecules in animals. Heme moieties and hemoglobin in the light-exposed retina absorb light, which leads to the release and production of gasotransmitters such as carbon monoxide (CO) and nitric oxide (NO), and a downstream antidepressant effect^9,10^. CO itself serves as a critical cofactor linking the circadian-clock system with metabolism and behaviors^11^. Blood-borne gasotransmitters drain with the retinal venous blood to the cavernous sinus (CS). The veins of the CS enwrap the internal carotid artery, which create a unique anatomical location in which an artery travels completely through venous structures. These gaseous transmitters diffuse across the microscopically thin membrane of the CS into the internal carotid artery where they would otherwise be present at a significantly lower concentration and provide a humoral signal of daylight to the brain. This facilitates transfer of CO and NO from the ophthalmic venous blood to the core arterial blood, which bypasses possible dilution in the general circulation of the blood through the heart and the rest of the body.

Preliminary support for the photochemical effects upon CO concentrations proposed in this model has been demonstrated in hybrid pig–boars and pigs^12–14^. For example, ophthalmic venous blood during the longest photoperiod days of the year (near the summer solstice) has been demonstrated to show nearly three times higher concentrations of CO than during night or during the shortest photoperiod days of the year (near the winter solstice). Countercurrent transfer of various molecules (including dopamine and reproductive hormones) from venous to arterial blood has been demonstrated in the CS, which lends further plausibility to this model^15–17^.

There are at least two credible (and complementary) photobiological mechanisms for the observation of elevated CO measured in retinal venous blood after bright-light exposure. One is the photodissociation of CO bound to heme iron groups in a reaction discovered in the nineteenth century by Haldane and Lorrain Smith^18^. A second mechanism would be the well-documented stimulation by bright light of heme oxygenase (HO)^19^. Photostimulation simultaneously dissociates bound CO from the heme moiety of HO and activates the enzyme itself to produce more CO.

Using state-of-the-art techniques for precise quantitative measurement of CO production, we demonstrate the robust elevation of peripheral free blood CO concentrations after exposure to bright light of 10,000 lux illuminance, a standard brightness used in the treatment of depression. The demonstration of this robust and reproducible effect of light upon blood in the generation of CO, an intrinsic central nervous system gasotransmitter, supports possible humoral mechanisms and mediators of light’s antidepressant effects.

## Materials/subjects and methods

### Study population

Nonpregnant, nonsmoking healthy women 18 years of age or older were recruited from the ASHER (Assessing Stress, Health, Emotion, and Regulation) Registry Database of the Asher Center for the Study and Treatment of Depressive Disorders. The ASHER Registry was established to investigate clinical phenotypes and biomarkers and based within an urban outpatient women’s mental health clinic at Northwestern University. All subjects reported regular sleep-onset time before 0100 and regular awakening time after 0400 and had no reported evidence of a sleep-wake phase disorder. The study was approved by the Northwestern University Institutional Review Board and subjects provided written informed consent.

### Sample collection

In two similar studies, blood was drawn in the Northwestern Memorial Hospital Clinical Research Unit between 0830 and 1600 under ambient lighting of ~40 lux. This level of illuminance is generally considered to be dim light. By contrast, illuminance considered “bright light” treatment for SAD is typically in the range of 2500–10,000 lux. Blood specimens of ~4.5 ml were drawn into light-protected aluminum foil covered 5 cc lithium-heparin tubes. Immediately after collection, tubes were gently inverted 8–10 times, had their exteriors decontaminated and were then placed on ice and sent to the laboratory for processing. Under the light of <50 lux in the laboratory, blood was transferred from each collection tube to corresponding 20 cc headspace vials whose caps were immediately crimp-sealed after blood transfer. “Headspace” measurements are the research standard method for analyzing volatile and semivolatile components of a liquid sample by sampling the air above the liquid surface. Crimp-top vials are generally accepted as providing containment for small stable gases such as CO. Each vial with cap was weighed before and after blood transfer in order to determine the exact mass transferred into the vials. Separate empty sealed vials were simultaneously exposed to ambient air at the beginning and end of each day’s blood sampling for later measurement of baseline CO in the lab. Variation was minimal and the average CO concentration in the ambient air was subtracted later from the observed test measurements to quantify blood-associated concentrations of CO. Each vial was wrapped in aluminum foil to shield it from light exposure.

In the first study, samples were collected over 2 days and vials were stored in a refrigerator set at 4 °C and shipped after the second day in a cold pack at the same temperature to Aspen Research Corporation (Maple Grove, Minnesota) for CO analysis. The next morning the vials were removed from refrigeration and allowed to warm to room temperature over 30 min. The foil from one of each pair of blood-containing vial was removed and that vial was gently mechanically rolled and exposed to 10,000 lux bright white light from a Carex Day-Light Classic light box at 12 in. distance. (Foil was left on the other tube to provide maximum shielding of any potential ambient light exposure.) Rolling of the tubes was done in part to reduce the risk of clotting, and especially to circulate the blood within the tube assuring consistent and complete exposure of the blood to the light. If the tube was stationary, only the blood on the perimeter of the tubes would get direct light exposure. Ambient temperature at the position of the vials was not affected by the light exposure. To serve as a control, the other vial had the same procedure without the mechanical rolling and without the foil being removed. A duration of 2 h of light exposure (or darkness) was used to replicate 10,000 lux light effects upon blood CO in a similarly designed prior study of pigs (unpublished observation Dan A. Oren, Maria Romerowicz-Misielak, Katarzyna Kozioł, Marek Koziorowski). After 2 h, 1 cc of headspace gas was withdrawn from each vial with a gas tight syringe and CO concentrations were measured in all vials using gas chromatography. No replicate lab measurements were performed. Ambient vial CO concentrations were subtracted from the two blood CO concentrations.

The second study was conducted to be certain that any difference between the active and control conditions was not due to motion in the light-treatment condition. This study was conducted in a similar fashion except that the unexposed vials also received mechanical rolling prior to CO analysis. The other vial underwent the same procedure without the foil being removed as a control.

To increase sensitivity for CO, samples were analyzed using an Agilent 6890 GC/FID interfaced to a modified PolyArc® methanizer. The PolyArc® system catalytically converts molecules containing carbon (in this case CO) to methane which is then detected by a flame ionization detector. Chromatography occurs prior to the methanization. The column used was an Agilent HP-Plot Q (Agilent 19095-Q04E, 30 m × 0.53 mm × 0.04 mm). Inlet temperature was 120 °C. Total helium flow was 18.1 ml/min. Injection volume was 1 ml from a manual gas tight syringe. Hydrogen to Flame Ionization Detector flow was 4.0 ml/min. Air flow was 350 ml/min. Makeup flow (constant He) was 23.0 ml/min. Flame Ionization Detector temperature was 300 °C. PolyArc® temperature was 293 °C. PolyArc® air pressure was 15 psi (11.5 ml/min). PolyArc® hydrogen pressure was 21 psi (46 ml/min). In order to reduce the noise effect on the CO from the dead volume elution, the column was started at a subambient temperature. This required modifying the PolyArc® to eliminate condensation of water from the PolyArc® exit line to the FID. Standards were prepared by purging 20 cc headspace vials with helium and immediately capping them with a septum. Spikes of known volumes of a standard 250 ppm CO stock gas were made to the 20 cc vials to yield standards of CO analyzed per the conditions above.

Lab personnel determining the CO concentrations were blinded to the mass of each sample analyzed. Lab-reported concentrations of CO were then independently mass normalized by dividing the reported concentration by the mass of each sample to determine a concentration expressed in parts per million per gram (ppm/g).

### Statistics

In both studies, two-tailed paired *t*-tests were planned comparing CO concentrations for each subject’s sample unexposed to bright light vs. that subject’s sample with bright-light exposure. A paired *t*-test is generally considered robust to different variances and degrees of normal variation between the two comparison groups. In post hoc analysis for each study, we performed a simple linear regression to explore potential associations between time of day of sample collections and degree of increase of CO concentrations. Both sets of statistics were calculated with GraphPad Prism version 8.30 for Macintosh.

Because of robust and consistent demonstrations of bright light’s effects in the aforementioned study in pigs, we were confident that if a similar phenomenon existed in human blood, a sample size of ten would more than suffice to demonstrate the expected acute effect of light. Based on our results from our own first study, we had a power of 99% for detecting the mean level of differences that we observed at *p* < 0.05 (two sided) in differentiating CO effects of light vs. dark. Based on our robust results from that first study, we used a smaller sample of 11 in the second study, which still had a power of 99% for detecting the same level of differences as in the first study^20^.

For the linear regression analysis exploring association between time of day and degree of light effect, with 24 in the first study we had a power of 79% to detect a large effect size of 0.59 at *p* < 0.05. Based on the results in the second study study linear regression, with only 11 subjects, we had a power of 42% to detect a large effect size of 0.59 at *p* < 0.05^21^.

## Results

### Effects of bright light

**Fig. 1:**
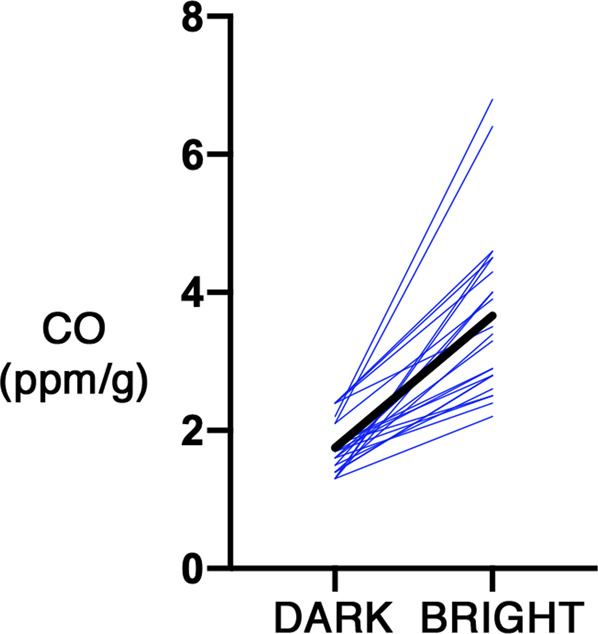
Thin lines depict individual CO concentrations in the first study. The thick line depicts mean values from the two conditions.

In the first study, after samples of blood were taken from 24 women (mean age = 42 ± 14 years), we found that light-unexposed peripheral blood headspace gas had mean CO concentration of 1.8 ± 0.4 SD ppm/g. Blood samples exposed for 2 h to bright white light of 10,000 lux had mean CO concentration of 3.7 ± 1.2 ppm/g (*p* < 0.0001, two-tailed paired *t*-test, *t* = 8.728, df = 23) (Fig. 1).

**Fig. 2:**
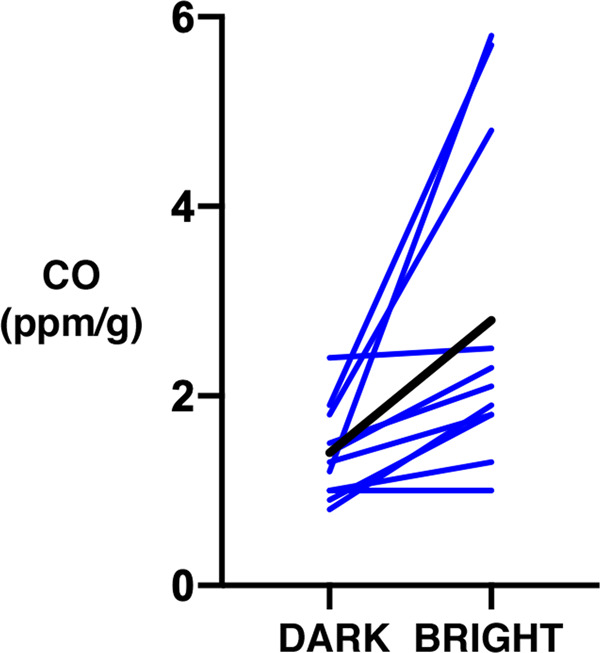
Thin lines depict individual CO concentrations in the second study. The thick line depicts mean values from the two conditions.

In the second study, after samples of blood taken from 11 women (mean age = 46 ± 13 years), light-unexposed peripheral blood headspace gas had mean CO concentration of 1.4 ± 0.5 SD ppm/g. Identically treated blood samples with 2 h of exposure to bright white light of 10,000 lux had mean CO concentration of 2.8 ± 1.7 ppm/g (*p* < 0.02, two-tailed paired *t*-test, *t* = 2.966, df = 10) (Fig. 2).

### Association of time of day with bright-light effect

**Fig. 3:**
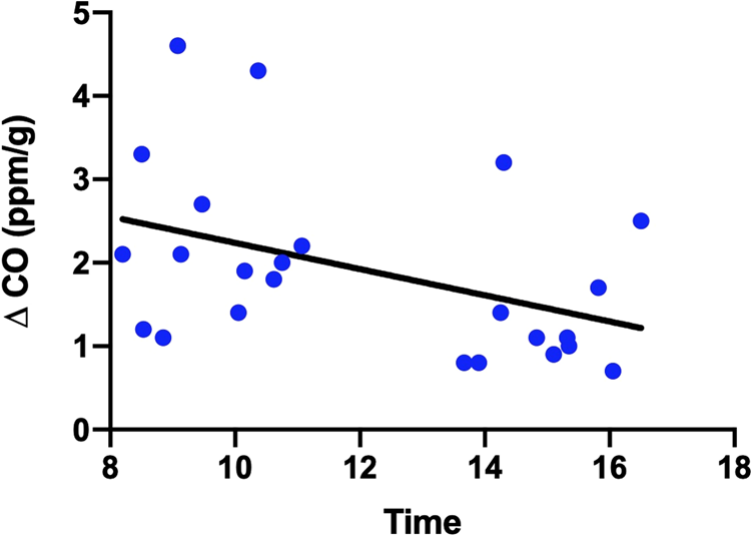
Dots depict increase in CO concentration associated with light exposure for each individual in the first study. The line depicts a “best-fit” curve from linear regression.

In the first study of 24 women, during the daytime, time of day was significantly inversely associated with increase in CO concentration under bright light vs. dark (*F*(1,22) = 4.843, *p* < 0.04), with an *R*^2^ of 0.1804 (Fig. 3).

**Fig. 4:**
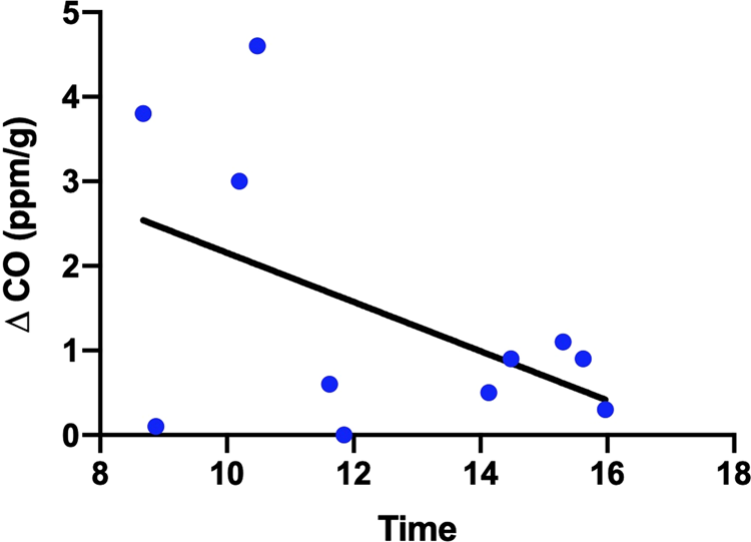
Dots depict increase in CO concentration associated with light exposure for each individual in the second study. The line depicts a “best-fit” curve from linear regression.

In the second study of 11 women, during the daytime, time of day was visually, but not significantly, inversely associated with increase in CO concentration under bright light vs. dark (*F*(1,9) = 2.961, *p* = 0.12), with an *R*^2^ of 0.2476 (Fig. 4).

## Discussion

Bright light of 10,000 lux illuminance (comparable to ambient daylight) robustly elevated free CO concentrations in blood and detectable in ambient gas of blood specimens. One likely mechanism is the catalytic release by the light of CO bound to hemoglobin. Although this biochemical release was first documented over a century ago, our data confirm the finding with state-of-the-art chemical techniques. This light-induced chemical dissociative process was dismissed at its discovery as clinically irrelevant and later ignored due to the mistaken belief that CO was biologically inert, except as a poison that interfered with normal hemoglobin oxygen-carrying capacity. Other scientists have erroneously hypothesized that any free CO would be immediately scavenged by hemoglobin.

The research technique we used of gas chromatography flame ionization detection of headspace air is the “gold standard” for assessing levels of free CO in a blood sample. This procedure is distinct from the routine measurement of carboxyhemoglobin saturation done in cases of suspected CO poisoning. That saturation level is the proportion of oxygen-binding sites on hemoglobin that instead of being occupied by oxygen are instead bound tightly to CO. Instead, we measured the level of free (unbound) CO in the blood indirectly by sampling the level of it in the headspace air above the blood in the tube. As there is presumably an equilibrium between the free levels of CO in the liquid and the air in the tube, the headspace concentration is a reliable measurement of the free CO levels in blood. Free CO is a gasotransmitter and therefore available for other chemical reactions.

The elevated concentrations of CO in the ambient gaseous space above the blood in the vial (headspace) in these studies demonstrate that CO released by blood is not immediately rebound to heme and has the potential to diffuse to act in tissues beyond the blood itself. The discovery reported 25 years ago that bright light stimulates the production by HO of CO offers an additional potential mechanism for the observation made in our studies^19^. The doubling of ambient CO concentrations by bright light suggests that even though scavenging of free CO by hemoglobin may be taking place, that effect is less than the combined potential effects of: (1) light-induced release of CO from hemoglobin, and (2) production of new CO molecules. Therefore, bright light robustly increases net levels of free CO available for subsequent chemical effects.

In this set of in vitro studies, we documented that neither the lapse of 2 h of time nor the gentle rolling of the blood samples accounted for the doubling of ambient CO observed when bright light was shined on the blood samples. This eliminates the possibility that the finding observed in the active treatment condition of these studies was due to time or motion alone.

Although these studies were not designed specifically to examine the effect of time of day upon light’s effects upon blood CO concentrations, in post hoc analysis we found a significant inverse correlation between time of day (in the daytime) and degree of CO elevation associated with bright light in the first study, and a nonstatistically similar pattern in the second study, whose sample size was less than half of the first. One interpretation of this observation may be connected to circadian rhythms of physiological CO concentrations in peripheral blood samples. Although we know of no studies in humans specifically examining circadian rhythms of CO concentrations in peripheral blood, there are at least two studies in humans that have carefully documented a diurnal variation in serum bilirubin levels, with bilirubin levels peaking at the end of the night, and then declining during the day^22,23^. As bilirubin production in mammals is a surrogate for CO production (as they are both produced virtually simultaneously in the same chemical pathway producing bilirubin’s precursor, biliverdin, and CO), it is probable that CO concentrations in blood would similarly peak at night’s end. Further, breath CO concentrations (an accepted surrogate marker for bilirubin production) also have a circadian rhythm that peaks at the end of the night^24^.

The finding that light has a greater effect in CO production earlier in the day is consistent with ample literature that light therapy for SAD is often more effective in the early morning, as opposed to later in the day^25^. In bipolar disorder early morning light may be overly effective and pose a risk for inducing of mania^26^. The rationale for why early morning light is generally more effective than light later in the day has been suggested to be related to its capacity to shift circadian rhythms earlier, which mid-daylight will not do. This study does not address that question. This study suggests (with limitations that only a few hours of the day were sampled) that light’s effect upon increasing CO levels in the blood also tends to be strongest in the early hours of the day. Physiologically our bodies normally produce their greatest amounts of CO right at the end of the night, driven by HO. Perhaps part of the strong effect of early morning light is that it also is used right at the time of day when light is the most effective at producing CO? This may add further to the rationale for considering the role of CO in light’s effects in affective disorders.

Our study population was female, so these findings remain to be confirmed in males, although there is no a priori reason to expect that there will be a different result in men than in women. These results are still germane to understanding potential physical mechanisms by which light therapy has an antidepressant effect, in that, women tend to be more susceptible to depression than men and, especially during the reproductive ages and in northern latitudes, women also have higher rates than men of experiencing winter seasonal depression and subsyndromal seasonal depression (both responsive to light therapy)^27^.

While this finding does not prove any clinical effect of the physical phenomena, the robustness of the finding suggests that humoral mechanisms of visible light’s effects be contemplated. It is commonly recognized that CO binds to hemoglobin and is a competitive inhibitor that displaces the oxygen-carrying sites of hemoglobin. CO poisoning is a well-known fatal risk of exposure to elevated concentrations of CO. But the impression that CO is only a poisonous and toxic gas is outdated in the context of current understanding of physiology. For example, typical concentrations of blood CO in nonsmokers measure up to 1% saturation of oxygen-binding sites of hemoglobin. But this binding is not irreversible. Physiologically produced CO is carried by hemoglobin and diffuses out of the body through pulmonary gas transport via diffusion across the pulmonary blood capillaries into the alveoli. Especially important for numerous aspects of physiology, CO metabolism and production is critical to the function of circadian-clock machinery, metabolism and behavior through regulation of CLOCK-BMAL1-dependent circadian gene expression and glucose metabolism^11^. When CO is generated de novo or when it is released from hemoglobin—both processes robustly catalyzed by light—and can circulate in the bloodstream it becomes a potential physiological gasotransmitter, capable of acting in numerous locations, including circadian regulation. In contrast to the view often held by clinicians that CO is invariably a poison, it is a critical physiological regulator. Other critical physiologically active gases such as NO are also subject to dissociation from hemoglobin by bright light and to stimulation of production by bright light.

The chief limitation of this work in is that it was conducted in vitro, as opposed to in vivo. Samples were also analyzed at room temperature rather than at physiological temperature, although evidence that dissociation of CO from carboxyhemoglobin is promoted by higher temperatures^28^ suggests that the effect seen in vitro should be similarly demonstrable at physiological temperature. Nonetheless, the results are consistent with observations in an in vivo model of a pig–boar hybrid that significantly higher concentrations of CO are observed in the daytime in summer than at night or in winter in ophthalmic venous blood^12^.

Further in vivo trials of the humoral phototransduction model are needed. These preliminary results inspire investigations of such questions as what minimum duration of light exposure would be needed to demonstrate a robust change in CO concentrations, what intensity of light exposure is needed to demonstrate a robust change in CO concentrations, are specific wavelengths of light more effective than others at eliciting the CO response, is there truly a circadian rhythm of the responsivity of blood CO concentrations to light? Demonstration of clinical antidepressant effects of light in association with changes in ophthalmic blood CO concentrations and brain blood concentrations of CO are also required to test the clinical significance of these findings.

Many, if not most, practicing physicians have little awareness of the physiology of CO beyond its danger as a toxin from excess inhalation. Bright summer daylight is associated with physiological increases in blood CO levels in retinal ophthalmic vessels^10,12–14^. The magnitude of increase in CO concentrations is comparable to that observed in the physiological variation in retinal ophthalmic blood CO levels after bright summer light in an animal model and consistently less than levels considered toxic in humans^12,29^. Numerous blood-borne chemicals (potassium and glucose are prominent examples) are critical regulators of physiology yet become toxic when present in excess amounts. CO should be considered similarly. An increasing body of knowledge over the past 25 years has established a wide range of critical physiological processes regulated in part by CO and by the endogenous production of CO^30^. In view of the robust effect of bright white light upon CO physiology, consideration of the broader effects of light exposure upon human physiology is compelling.
